# Is population structure sufficient to generate area-level inequalities in influenza rates? An examination using agent-based models

**DOI:** 10.1186/s12889-015-2284-2

**Published:** 2015-09-23

**Authors:** Supriya Kumar, Kaitlin Piper, David D. Galloway, James L. Hadler, John J. Grefenstette

**Affiliations:** Department of Behavioral and Community Health Sciences, Graduate School of Public Health, University of Pittsburgh, 704A Parran Hall, 130 DeSoto Street, Pittsburgh, PA 15261 USA; Department of Biological Sciences, University of Pittsburgh, Pittsburgh, PA USA; Public Health Dynamics Laboratory, Graduate School of Public Health, University of Pittsburgh, Pittsburgh, PA USA; Emerging Infections Program, Yale School of Public Health, Yale University, New Haven, CT USA; Department of Health Policy and Management, Graduate School of Public Health, University of Pittsburgh, Pittsburgh, PA USA

**Keywords:** Agent-based model, Influenza inequalities, Area-level inequalities, Census tracts, Poverty

## Abstract

**Background:**

In New Haven County, CT (NHC), influenza hospitalization rates have been shown to increase with census tract poverty in multiple influenza seasons. Though multiple factors have been hypothesized to cause these inequalities, including population structure, differential vaccine uptake, and differential access to healthcare, the impact of each in generating observed inequalities remains unknown. We can design interventions targeting factors with the greatest explanatory power if we quantify the proportion of observed inequalities that hypothesized factors are able to generate. Here, we ask if population structure is sufficient to generate the observed area-level inequalities in NHC. To our knowledge, this is the first use of simulation models to examine the causes of differential poverty-related influenza rates.

**Methods:**

Using agent-based models with a census-informed, realistic representation of household size, age-structure, population density in NHC census tracts, and contact rates in workplaces, schools, households, and neighborhoods, we measured poverty-related differential influenza attack rates over the course of an epidemic with a 23 % overall clinical attack rate. We examined the role of asthma prevalence rates as well as individual contact rates and infection susceptibility in generating observed area-level influenza inequalities.

**Results:**

Simulated attack rates (AR) among adults increased with census tract poverty level (F = 30.5; *P* < 0.001) in an epidemic caused by a virus similar to A (H1N1) pdm09. We detected a steeper, earlier influenza rate increase in high-poverty census tracts—a finding that we corroborate with a temporal analysis of NHC surveillance data during the 2009 H1N1 pandemic. The ratio of the simulated adult AR in the highest- to lowest-poverty tracts was 33 % of the ratio observed in surveillance data. Increasing individual contact rates in the neighborhood did not increase simulated area-level inequalities. When we modified individual susceptibility such that it was inversely proportional to household income, inequalities in AR between high- and low-poverty census tracts were comparable to those observed in reality.

**Discussion:**

To our knowledge, this is the first study to use simulations to probe the causes of observed inequalities in influenza disease patterns. Knowledge of the causes and their relative explanatory power will allow us to design interventions that have the greatest impact on reducing inequalities.

**Conclusion:**

Differential exposure due to population structure in our realistic simulation model explains a third of the observed inequality. Differential susceptibility to disease due to prevailing chronic conditions, vaccine uptake, and smoking should be considered in future models in order to quantify the role of additional factors in generating influenza inequalities.

**Electronic supplementary material:**

The online version of this article (doi:10.1186/s12889-015-2284-2) contains supplementary material, which is available to authorized users.

## Background

Health inequalities, or the differential experience of health between subpopulations, pose major challenges for the control and prevention of illnesses. People of low socioeconomic status (SES) and people in high-poverty neighborhoods experience many worse health outcomes than higher SES populations [1]. In the case of transmissible infections, researchers have shown a link between SES and respiratory infections including influenza [2–8]. At the household-level, income and educational level are strong predictors of respiratory infection [8–10]. Recent studies have shown a strong, persistent link between area-level poverty and influenza hospitalization rates. High-poverty census tracts in New Haven County, CT (NHC) had significantly higher influenza hospitalization rates during multiple influenza seasons compared to lower-poverty tracts [9, 10]. In New York City, influenza hospitalization rates during the first wave of the 2009 H1N1 pandemic were significantly higher in high-poverty areas than in low-poverty areas [11].

Household- and area-level density are correlated with higher disease rates [3, 9], but whether these factors are sufficient to generate the observed area-level inequalities in influenza remains unknown. In NHC, influenza hospitalization rates were correlated not only with the poverty level, but also with crowding in census tracts [9]. On the other hand, an analysis of 305 administrative units in the UK suggested that population density and household crowding were insufficient to explain differential influenza rates during the 1918 influenza pandemic [12], and an analysis of influenza mortality in 10 US cities suggested that population density was only weakly correlated with mortality during the 1918 pandemic [13]. It remains unclear if population structure (population density and age-structure) is sufficient to generate the spatially differentiated patterns of influenza hospitalization and mortality that have been observed in surveillance data.

The Connecticut Emerging Infections Program (CT-EIP) conducts surveillance for hospitalized, lab-confirmed influenza cases. The program geo-codes each case to its residential census tract, allowing an analysis of influenza hospitalization rate by census tract poverty levels. This surveillance showed that adult and child influenza hospitalization rates increased as the proportion of people living below the federal poverty level in census tracts increased [9, 10]. This relationship was seen in multiple years from 2007 to 2011, but the mechanism that generates the unequal pattern of disease remains unknown. Because the distribution of household income differs by neighborhood poverty, exposure or susceptibility differences by household income may manifest as area-level inequalities by census tract poverty.

Differential exposure to virus, susceptibility to disease, and access to healthcare have been proposed as possible mechanisms that could generate unequal levels of influenza illness and death [5, 14–16]. If, in fact, higher hospitalization rates are a reflection of higher incidence rates, differential exposure due to larger household size, greater population density, and younger age-structure could explain unequal hospitalization rates. Attack rates were higher in households classified as lower SES in multiple cities during the 1918 influenza pandemic [17]. Community-based surveillance for acute respiratory infection in Tecumseh also suggested a higher incidence rate correlated with lower household income [8].

Once exposed, differential susceptibility due to differential vaccination, chronic condition prevalence, and smoking rates could further impact incidence rate inequalities [14]. Vaccination and smoking rates differ by household income [18, 19], as do rates of chronic conditions such as obesity [20], diabetes [21], and asthma [22]. In NHC, hospitalized cases from high-poverty tracts had lower vaccination rates and higher rates of chronic conditions [9], suggesting that cases were more susceptible to disease in these tracts. Alternatively, hospitalization rate inequalities could be manifestations of differential disease severity due to underlying chronic conditions and delays in healthcare access.

With so many potential drivers of inequalities, it is important to first ask if population structure is sufficient to generate the observed inequalities. Because influenza is typically documented only when ill people contact the healthcare system, i.e. in only a subset of cases, influenza surveillance data are not ideal to test if population structure is sufficient to generate differential incidence rates that reflect the magnitude of observed hospitalization rate inequalities. To address this question, we require innovative methods such as spatially explicit agent-based models.

Agent-based models using populations of agents with realistic spatial and demographic characteristics can serve as a counterfactual laboratory in which to test competing hypotheses regarding the possible causes of influenza inequalities [23, 24]. Here, we ask if population structure is sufficient to generate the observed inequalities in NHC influenza rates by area-level poverty. By population structure, we mean population density, age structure, and average household size in the residential census tract; and contact rates in each location (workplace, neighborhood, school, household). We use simulation models that include demographic factors (age structure, household size, and population density) and neighborhood, household, school, and workplace mixing to address this question. We present a temporal analysis of attack rates by poverty, and a validation of model findings using CT-EIP data. Agent-based models are especially useful in examining the role of individual-level rules in generating population-level patterns. In this case, we examined whether individual contact rates or household income-based infection susceptibility were able to generate the pattern of area-level disparities observed in NHC. We discuss our findings in relation to factors that could lead to differential area-level influenza rates and propose additional hypotheses for future research.

## Methods

### Synthetic populations and agent-based models

We use a synthetic population of NHC [25] and the agent-based modeling platform, FRED (Framework for Reconstructing Epidemiologic Dynamics) [26], to simulate influenza attack rates across census tracts. FRED is available open-source from http://fred.publichealth.pitt.edu/, and has been described in detail elsewhere [26]. FRED has been used in previous studies to evaluate the impact of public health policies for influenza [27] and measles [28]. Synthetic populations are representations of each individual and household in a geographic area of interest, with characteristics based on the American Community Survey (ACS), Integrated Climate and Land Use Scenarios (ICLUS) data, and the US census (see Additional file 1 for a summary) [25]. Agents—representing people—have socio-demographic characteristics including age, sex, race, employment status, household income, and household location. Each house, school, and workplace is mapped to a specific geo-location, reflecting its actual spatial location. Agents are assigned to schools or workplaces based on age, location, size of schools/workplaces, and commuting patterns [29]. Each agent in FRED is associated with health information including current health status, date of infection, infectiousness, and susceptibility [26, 27, 30–33]. Agent susceptibility refers to the probability of becoming infected (either asymptomatic or symptomatic) once exposed.

### Correlation between synthetic population and American Community Survey

In order to examine if the synthetic population is a good representation of real populations in NHC, we determined the correlation between the two populations. We calculated Pearson correlation coefficients between synthetic population-estimates and 2007–11 ACS-estimates of average household size, population density (number of people per square mile), and percentage of the population aged below 18 years (percent <18y) or 65 years and above (percent >=65y) in each census tract.

### Disease simulation using FRED

During each simulated day, agents interact with other agents who share the same social activity locations and have a probability of disease transmission (given contact) based on influenza natural history parameters [34] as reported previous studies [27, 33, 35–37]. See Additional file 1: Tables S1-S3 for these parameters. The epidemic was seeded with 100 infectious individuals (randomly selected from 856,971 simulated individuals in NHC) on Day 1. The time horizon for each simulation was 100 days. We simulated the epidemic incorporating age-based residual immunity (as observed before the 2009 H1N1 pandemic) [38] and compared results with simulations assuming complete susceptibility in the population. Inequalities between high- and low-poverty tracts did not differ in the two scenarios, so we report results from simulations assuming complete susceptibility to infection except when sweeping over a susceptibility modifier described below. Contact-numbers were calibrated to result in place-specific proportions of infections as follows: 30 % in households and 70 % outside, the latter including 33 % in neighborhoods, and 37 % in schools and workplaces [26, 35, 39, 40]. Daily contact-rates are shown in Additional file 1: Table S2 and transmission probabilities between individuals in Additional file 1: Table S3. In contrast to previous models, contact rates in neighborhoods were sensitive to neighborhood population density (people/unit area), but constrained to result in 33 % attack rate in the neighborhood.

Simulations were run 50 times and average attack rates were calculated for each census tract. Attack rates were calculated as the cumulative number of infections per 100 adults over the course of the epidemic. For a baseline simulation, we used a viral transmissibility that resulted in a simulated clinical attack rate (AR_SIM_) of 23 % in NHC—similar to the observed rate of 21 % in Connecticut during the 2009 H1N1 pandemic based on a hospitalization rate of 35.2/100,000 and published multipliers to estimate an incidence rate [41, 42]. We also swept over transmissibility values to result in AR_SIM_ values of 33 and 40 % in order to simulate epidemics caused by viruses of higher transmissibility [43].

### Analysis by census tract poverty

Census tracts were categorized into four groups (0–4.9 %, 5–9.9 %, 10–19.9 %, > = 20 %) based on the percent of population that lived below the federal poverty line in the 2007–2011 American Community Survey (ACS). These categories are referred to as poverty level 1 (0–4.9 %), poverty level 2 (5–9.9 %), poverty level 3 (10–19.9 %), and poverty level 4 (> = 20 %) in the rest of this paper. We calculated a mean AR_SIM_ among adults or children for each census tract across 50 FRED runs, and then generated a mean AR_SIM_ and 95 % confidence interval over all tracts in each poverty level. We used a oneway ANOVA to find the F-statistic and the associated p-value to test the null hypothesis that the mean ARs in each of the 4 poverty levels were the same. To calculate 95 % CIs, we used the mean and standard error of census tract ARs in each poverty level: 95 % CI = mean +/− 1.96 (SE). We used a Bonferroni correction for multiple comparisons to calculate significance values for pairwise differences in mean AR between poverty levels. To calculate attack rate ratios from our simulations (RR_SIM_), we divided cumulative incidence rates at the end of the epidemic (AR_SIM_ on day 100) at each higher poverty level by the AR_SIM_ on day 100 in poverty level 1. Using the number of hospitalized cases among adults in each NHC census tract from CT-EIP data (cases geocoded to census tracts based on boundaries established during the 2010 decennial census) and the population in tracts based on the 2010 census, we calculated expected attack rates (AR_EIP_) by poverty level using previously published multipliers that account for underreporting H1N1 influenza hospitalizations and influenza incidence in the population [42] (i.e., we multiplied the number of reported hospitalizations in each poverty category by 2.7 to arrive at a corrected number of expected hospitalizations and further multiplied this number by 221.79 to estimate the number of infections among adults). To calculate attack rate ratios from EIP data (RR_EIP_), we divided AR_EIP_ at each higher poverty level by the AR_EIP_ at the poverty level 1. We calculated relative risk 95 % confidence intervals around these point estimates of the rate ratios [44].

To examine hospitalization rates over time from the CT-EIP data, we divided lab-confirmed influenza-associated hospitalized case counts from tracts in each poverty level by the total population in each poverty level and plotted daily rates per 100,000 people between Oct 1 2009 and April 30 2010. Analysis of de-identified data from New Haven County was deemed to not include involvement of human subjects by the University of Pittsburgh Institutional Review Board.

### Asthma prevalence

We conducted post-hoc analyses of simulation output to examine the impact of differential asthma prevalence by poverty and higher influenza hospitalization rates among asthmatics on expected hospitalization rates at each poverty level. Asthma prevalence among those below the poverty line and those above was determined based on data from the National Health Interview Survey (2006–2008): 11 % of those below the federal poverty line and 7.3 % of others were assumed to be asthmatic [45]. We assumed the same attack rate for asthmatic and non-asthmatic people to calculate AR_SIM_ among asthmatic and non-asthmatic adults. In CT-EIP data [9], we observed that the average annual influenza hospitalization rate among asthmatic adults was 2.7 times that among non-asthmatics. We calculated predicted hospitalization rates/100,000 adults from the simulation [42] and multiplied the predicted hospitalization rate among asthmatics by this factor, thus allowing us to estimate hospitalization rates among adults while accounting for asthma prevalence.

### Sensitivity to neighborhood contact rates and income-based susceptibility

In order to examine whether an increased rate of contact in the neighborhood was sufficient to generate the observed area-level disparities, we systematically varied the neighborhood contact rate value by 10 % increments between 20 % lower and 50 % higher than the calibrated neighborhood contact rate value (i.e. 8 values). All other parameters were held constant and only the neighborhood contact rate value was varied in order to examine its independent impact on attack rate disparities. We performed 50 runs with each parameter setting and calculated the rate ratio between tracts in poverty level 4 and tracts in poverty level 1.

In order to examine the significance of household income-based differential influenza susceptibility in generating the observed area-level inequalities, we gave agents a susceptibility modifier between 0 and 1. Susceptibility had a linear relationship with household income. We examined the sensitivity of model output to a range of modifier values such that the highest-income households had between 91 and 14 % of the susceptibility of lowest-income households. Because the household income distribution has a long tail (range between $150,000 and 1.3 million in the highest tenth decile), all households above the 90th percentile of household income had the same susceptibility modifier value. We systematically changed the susceptibility modifier value to determine the household-income-related differential susceptibility that would be required in order to generate the area-level differential influenza rates observed. As before, we performed 50 runs with each parameter setting and calculated the rate ratio between tracts in poverty level 4 and tracts in poverty level 1 (see Additional file 1).

All simulation raw data and analysis is available online at http://fred.publichealth.pitt.edu/publications.php

## Results

### High correlation between synthetic population and American Community Survey (ACS)

We tested the correlation between synthetic population-estimates and ACS 07-11-estimates of demographic distributions to examine if the synthetic population reflected reality accurately. As shown in (Additional file 1: Figure S1), Pearson correlation coefficients between the synthetic population and the ACS were 0.86 (percent of the population in each tract that was <18y), 0.88 (Average household size in census tracts), 0.95 (Percent adults 65y and above in tracts), and 0.99 (population density in census tracts).

### Simulated attack rates increase with census tract poverty

We simulated an epidemic with an overall AR_SIM_ of 23 % in NHC. Though there are orders of magnitude differences between AR and hospitalization rates, the epidemic curve from our simulation is comparable to the epidemic curve using hospitalization rates from surveillance (see Additional file 1: Figure S2).

**Fig. 1:**
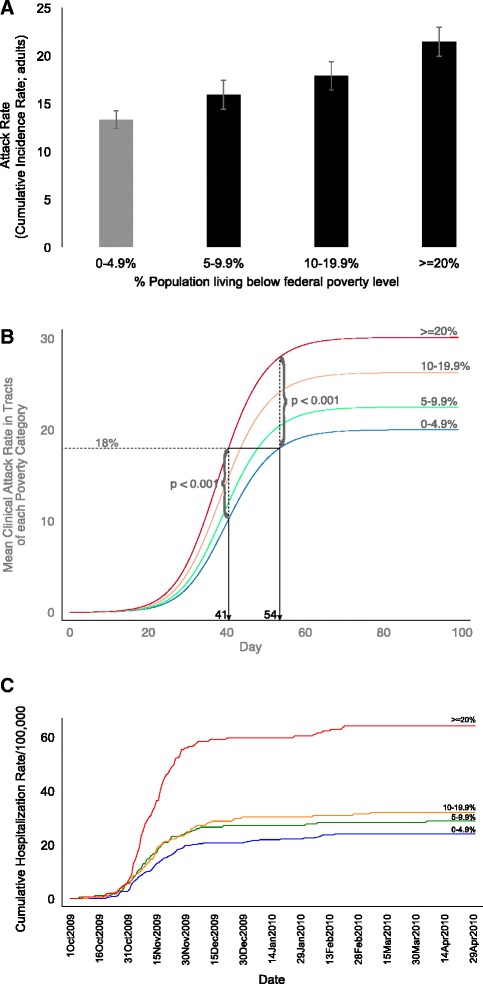
Mean number of infections per 100 adults in census tracts within each poverty category. Error bars signify 95 % CI. (**a**) Time course of epidemic in tracts of each poverty level. Mean simulated clinical attack rate (cumulative incidence rate) over tracts in each poverty level is plotted against epidemic day. 18 % AR_SIM_ (90%of the maximum AR_SIM_ in lowest-poverty tracts) is shown (**b**). Cumulative hospitalization rate in tracts of each poverty level is plotted against date between Oct 1 2009 and Apr 30 2010 (**c**)

AR_SIM_ among adults (measured as the cumulative incidence among 100 adults at the end of the epidemic) increased as poverty increased in the census tract (F = 30.54, *P <* 0.001) (Fig. 1a). Adult AR_SIM_ was significantly different between poverty level 1 and each of poverty level 2 (*P* = 0.025), poverty level 3 (*P <* 0.001), and poverty level 4 (*P* < 0.001). AR_SIM_ was also significantly different between poverty level 2 and poverty level 4 (*P* < 0.001), and between poverty level 3 and poverty level 4 (*P* = 0.002), but not between poverty levels 2 and 3 (*P* = 0.278). Population density increased with poverty level of census tracts, and was significantly related to the mean clinical attack rate among adults in multivariable regression models (see methods, in Additional file 1: Figure S3, and Table S4).

AR_SIM_ among children ranged from 42.00 % in poverty level 1 to 45.71 % in poverty level 4. Rates were significantly different between poverty levels (F = 15.30; *P* < 0.001). In this case, AR_SIM_ was significantly different between poverty level 1 and each of poverty level 3 (*P* < 0.001), and poverty level 4 (*P* < 0.001), but not poverty level 2 (*P* = 0.130). AR_SIM_ among children was also significantly different between poverty level 2 and poverty level 4 (*P* = 0.002). The difference in AR_SIM_ among children between poverty levels 2 and 3 (*P* = 0.604) and between poverty levels 3 and 4 (*P* = 0.283) were not significant.

### Higher poverty tracts have earlier increases in infection rates

Simulations allow us to track attack rate dynamics over the epidemic. We examined overall AR_SIM_ (for the entire population including children and adults) dynamics in each poverty level over the duration of the epidemic. As shown in Fig. 1B, tracts in poverty level 1 experienced an AR_SIM_ of 18 % (90 % of the maximum AR_SIM_ in these tracts) on day 54 of the epidemic, whereas poverty level 4 tracts had an AR_SIM_ of 18 % on day 41 of the epidemic. AR_SIM_ was significantly different (*P* < 0.001) between poverty levels 1 and 4 on both days 41 and 54. AR_SIM_ reached 23 % (the overall AR_SIM_ in NHC) on day 47 in poverty level 4 whereas tracts in poverty level 1 did not reach this AR_SIM_ at all.

The temporal lag between the highest- and lowest-poverty census tracts is an emergent property from the model, and we validated this outcome of the model by examining CT-EIP data on hospitalizations by census tract poverty level from the (H1N1) 2009 pandemic. As shown in Fig. 1C, high poverty tracts in NHC were in fact observed to have a steeper, earlier increase in hospitalization rate during the second wave of the (H1N1) 2009 pandemic. The temporal trend was similar in the first wave of the pandemic (not shown).

### Simulated attack rate ratios fall short of observed rate ratios

**Fig 2:**
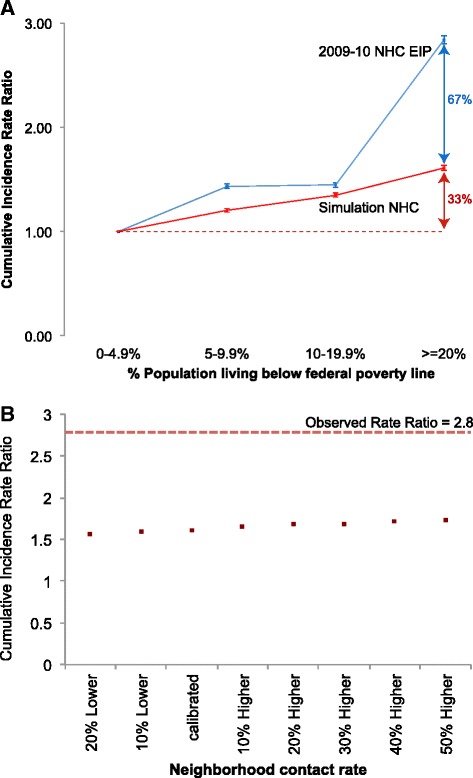
Incidence rate ratio between each higher poverty category and poverty level 1 (0–4.9 %). Blue: Emerging Infections Program data from NHC, 2009–2010. Red: simulation results for NHC (**a**). Sweep of the neighborhood contact rate. Dots represent the ratio of attack rates in the highest to lowest poverty census tracts (**b**)

We compared the magnitude of area-level inequalities generated by our model to that observed in CT-EIP data. Using poverty level 1 census tracts as our reference population, we compared RR_EIP_ (adults) from the 2009–10 CT-EIP to RR_SIM_ from our simulations. RR_SIM_ (red line, Fig. 2a) fell short of RR_EIP_ (blue line, Fig. 2a), most notably at poverty level 4. Our model generated 33 % of the rate difference (attributable risk) between the highest and lowest poverty census tracts from the CT-EIP data. The RR_SIM_ among children in each higher-poverty category compared to the poverty level 1 were 1.03, 1.06, and 1.09 respectively from our simulation. This represents a small fraction of the RR_EIP_ of 3.2 between poverty levels 4 and 1 in the CT-EIP.

### Accounting for asthma prevalence does not explain shortfall

Asthma prevalence rates differ between those living below the federal poverty line and those above the poverty line. We hypothesized that accounting for asthma prevalence and the differential probability of influenza hospitalization between asthmatics and non-asthmatics may reduce the shortfall in the RR_SIM_ compared to RR_EIP_. As shown in Table 1, however, the hospitalization rate increased only slightly when asthma prevalence was taken into account and fell short of RR_EIP_.

**Table 1:** Observed and predicted hospitalization rates/100,000 adults. Observed rates in New Haven County between Aug 2009 and Aug 2010, predicted hospitalization rate in each poverty category from our simulation and predicted rates after accounting for asthma prevalence rates are shown

% Population living below federal poverty line	Observed	Simulation	Sim + Asthma Prevalence^b^
	2009–10 Hospitalization Rate/100,000 (RR)^a^	Predicted Hospitalization Rate/100,000 (RR)^a^	Predicted Hospitalization Rate/100,000 (RR)^a^
0–4.9 %	20.91 (1.00)	22.16 (1.00)	24.89 (1.00)
5–9.9 %	29.99 (1.43)	26.50 (1.20)	29.83 (1.20)
10–19.9 %	30.25 (1.45)	29.82 (1.35)	33.70 (1.35)
> = 20 %	59.39 (2.84)	35.79 (1.61)	40.87 (1.64)

^a^RR Rate Ratio

^b^Hospitalization rates estimated from simulated attack rates taking into account asthma prevalence by poverty level

### Sensitivity to neighborhood contact rates and income-based susceptibility

Over a range of values for neighborhood contact rates from 20 % below to 50 % above the baseline value reported in Additional file 1: Table S2 (which was calibrated to produce a 33 % AR in the neighborhood and 30 % in households), we observed no appreciable change in the RR_SIM_ between the highest and lowest poverty tracts (Fig. 2B). At 20 % lower contact rates in neighborhoods, the proportion of all infections that occurred in the neighborhood and households was 26 and 31 % respectively. At 20 % higher contact rates in neighborhoods, the proportion of all infections that occurred in the neighborhood and households was 34 and 28 %, and at 50 % higher contact rate in neighborhoods, the proportions were 37 and 26 % respectively (see Additional file 1: Figure S4 in the additional file for a comparison of place-based infection proportions between baseline and 50 % higher contact rates in the neighborhood).

Individual susceptibility to influenza was varied based on household-income. We observed that when highest-income households were 14 % as susceptible as lowest-income households, RR_SIM_ equaled the observed RR_EIP_ (Additional file 1: Figure S5 and Table S5). The RR_SIM_ among children between poverty levels 4 and 1 was 1.63, representing 29 % of the children’s hospitalization rate difference observed in the CT-EIP data.

### Sensitivity of model results to transmissibility of virus

**Fig. 3:**
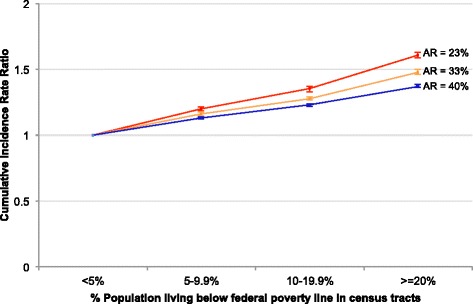
Sensitivity of rate ratios to transmissibility of the virus. Rate Ratios are shown for three simulated clinical attack rates: 23, 33, and 40 %

In order to examine the sensitivity of model results to virus transmissibility, we examined RR_SIM_ over a range of transmissibility values that resulted in AR_SIM_ ranging from 23 to 40 %. We observed that the RR_SIM_ between each higher poverty category and the lowest poverty category decreased as virus transmissibility increased (Fig. 3).

## Discussion

Though area-level inequalities in influenza hospitalization and mortality rates have been reported [9–12], the causes remain unclear. Knowledge of the causes and their relative explanatory power will allow us to design interventions that have the greatest impact on reducing inequalities. Spatially explicit simulation methods allow us to test the role of hypothesized social factors in generating area-level inequalities. We used agent-based models with realistic populations to test the sufficiency of population structure in generating the observed area-level inequalities in influenza hospitalization rates in NHC. An epidemic simulation model that included demographic factors (age structure, household size, and population density), as well as neighborhood, household, school, and workplace mixing, accounted for 33 % of the positive correlation between census tract poverty level and observed influenza rates. Higher poverty areas had not only higher simulated infection rates, but also earlier and steeper increases in infection rates—an emergent property of the model that we corroborated using surveillance data. To our knowledge, this is the first study to use simulations to probe the causes of observed inequalities in influenza disease patterns.

Other studies have reported the ability of simulation models to generate spatially patterned influenza rates, but did not compare their results to observed surveillance data. Stroud *et al.* found that simulated influenza attack rates in southern California census tracts were significantly correlated with average household size, the proportion of population 5-18y age, population density, and per capita income [46]. Dalziel et al. showed that a model based on intra-city mobility patterns of individuals was sufficient to generate unequal influenza rates between Canadian cities [47]. We need to compare their results to surveillance data from California and Canada in order to determine if model-generated patterns of influenza incidence rates are comparable to those observed in reality.

### Temporal trends in census tracts

Our simulation suggested that high-poverty areas reach higher attack rates earlier during an epidemic than do lower-poverty areas. We examined the CT-EIP data for temporal differences in hospitalization rates by census tract poverty and saw that indeed, the data qualitatively validate our model findings. This result has important implications for preparedness and planning. Interventions should be rolled out in high-poverty areas before low-poverty areas. During the (H1N1) 2009 pandemic, vaccine first became available in October [48]. If the epidemic progresses at different rates in high- and low-poverty areas, it becomes important to increase availability of vaccine in high-poverty areas first. Past models have shown that targeting vaccine to high-poverty counties when vaccine is limited could reduce disease overall [36]. The impact of targeting non-pharmaceutical interventions to high-poverty areas on reducing influenza inequalities should also be examined.

### Individual agent-parameters that generate area-level inequalities

In our baseline model, all agents were equally susceptible to influenza. Under these conditions, increasing the individual contact rates in neighborhoods to higher than the calibrated value did not increase the incidence rate ratio between high- and low-poverty neighborhoods. As we increase the contact rate in the neighborhood, the proportion of infections in the neighborhood goes up as expected, with a resulting decrease in the proportion of infections occurring in other locations (compared to the calibrated contact rates in our baseline scenario). Our models then do not reflect observations and past assumptions regarding place-based infection rates [39, 49], precluding a sensitivity analysis with a larger range of neighborhood contact rate values. We expect that attack rates result from contacts not only in the residential tract but also in workplaces, schools, and households. In addition, intensity of contacts is lower in neighborhoods compared to the intensity in other locations (workplaces, schools, households). The extent of disparity that the model generates is therefore an emergent property of the model.

A systematic variation in household-level differences in susceptibility suggests that when the highest income households were substantially less susceptible to influenza (14 % as susceptible as the lowest income households), our model generated the area-level inequality that has been observed in NHC. Researchers have reported differential incidence and susceptibility by household SES [8, 17]. The relationship between income and susceptibility may not, however, be linear, as we assumed in our model. In order to probe the relationship between income and susceptibility, models may need to incorporate more detailed data on how vaccine uptake and smoking behavior, as well as chronic disease prevalence differ with household income, and how these behavioral and biological factors modulate susceptibility to infection.

### The role of virus transmissibility

Our model suggests that area-level inequalities are more pronounced with lower transmissibility of the virus. This is consistent with previous studies reporting that differential attack rates due to population structure were more pronounced when simulating pathogens with lower transmissibility [47]. Based on overall hospitalization rates from the CT-EIP [41] and RR_EIP_ between the highest and lowest poverty category tracts in NHC [50], it appears that influenza seasons with lower overall hospitalization rate in the population correspond with higher RR_EIP_ (signifying higher inequalities) [9, 50]. A likely explanation is that the greater the transmissibility, the higher the attack rate in all poverty categories, including the lowest poverty tracts, leading to lower inequalities.

### Limitations of the model

The current model does not account for disease symptoms or severity. This may limit our ability to model hospitalization rate inequalities that result from unequal disease *severity* between high- and low-poverty neighborhoods even if attack rate differences do not completely reflect the inequality observed. Future studies should include more detailed models of individualized symptoms. [51] A determinant of disease severity is underlying chronic disease. Obesity and chronic conditions such as diabetes and asthma increase the risk of hospitalization due to influenza [52, 53]. Similarly, stress, which is higher in low-income populations [54], increases influenza severity once infected [55]. In post-hoc analyses, we found that taking into account differential influenza hospitalization rates between asthmatics and non-asthmatics explained only a small additional proportion of observed inequalities in adult AR_EIP._ Future studies should account for uncontrolled asthma and other chronic conditions, and stress.

In addition, neighborhood-level factors, such as access to healthcare, may interact with household income to result in differential delays in health care access for influenza symptoms. This would result in cases being more severe in high-poverty neighborhoods at the time they first contact the healthcare system. Low-income people (unemployed adults and those without insurance) as well as those with underlying chronic disease are more likely to delay seeking healthcare when they have influenza symptoms. [56] Differential healthcare-seeking behavior may thus add to differential attack rates to close the gap between observed and model-generated inequalities in influenza hospitalization rates in NHC.

These same factors may also increase model-generated inequalities in children’s AR_SIM_, which in our current model did not approach that observed in reality. On the other hand, the finding that inequalities in children’s AR_SIM_ fell short of that observed in NHC may point to a limitation in the modeled contact mixing structure among children. A strength of our simulated populations is that they include realistic school sizes and locations [29], but there is little literature on contact probabilities in schools based on school size. An important use of models is to guide additional data collection [24, 58]. Contact-mixing data in schools should be collected, and such information, if available, should be incorporated into ABMs in the future.

## Conclusions

Our agent-based model provides a counterfactual laboratory to test hypotheses for the production of inequalities. It is difficult to compare alternative hypotheses for the generation of influenza disparities using observational techniques because of the large number of factors that would need to be controlled for in order to make conclusions. For example, in order to test the hypothesis that population structure is sufficient to account for influenza attack rate disparities, we would need to factor in household size, the number and contacts of school-going children, employment status and workplace contacts of adults, and population density and contacts in neighborhoods, in addition to controlling for alternative hypotheses such as stress and health behaviors. Given the small number of clinically confirmed influenza cases and the dynamics of infectious agent spread, observational methods are not ideal for such studies. As pointed out by Epstein [58], and more recently by Marshall and Galea [24], agent-based models are especially well-suited to the exploration of area-level inequalities because of their characteristic features: heterogeneity of agents and spatially explicit agent interactions. We have utilized these features of agent-based models to provide the first test of a proposed theoretical mechanism (crowding or population structure) for the generation of inequalities in influenza disease.

Results from the current study suggest that population structure in our simulations accounts for 33 % of the observed area-level inequalities in adult influenza hospitalization rates, leaving 67 % of the inequality to be explained by other factors. Future models should quantify the capacity of individual behavioral and biological factors to generate influenza inequalities thus allowing us to prioritize interventions aimed at factors with the greatest explanatory power.

## Additional file

Additional file 1:
**Appendix for “Is population structure sufficient to generate area-level inequalities in influenza rates? An examination using agent-based models”.** (PDF 642 kb)

## Abbreviations

AR_SIM_: Simulated attack rates
AR_EIP_: Observed attack rates in surveillance by the Emerging Infections Program
CT-EIP: Connecticut Emerging Infections Program
RR_SIM_: Rate ratio of attack rates from the simulation
RR_EIP_: Rate ratio of attack rates from observed Emerging Infections Program data
NHC: New Haven County, CT
SES: Socio-economic status
ACS: American community survey
