# Supplementary material for: Is population structure sufficient to generate area-level inequalities in influenza rates? An examination using agent-based models
Source: BMC Public Health. 2015 Sep 23;15:947. doi: 10.1186/s12889-015-2284-2 (PMC4579639; doi:10.1186/s12889-015-2284-2)
Supplement: Additional file 1: — Appendix for “Is population structure sufficient to generate area-level inequalities in influenza rates? An examination using agent-based models”. (PDF 642 kb) [file 12889_2015_2284_MOESM1_ESM.pdf]

Is population structure sufficient to generate area-level inequalities in influenza rates? An examination using agent-based models.

Synthetic population generation and data sources: See Wheaton, W.D., 2014. "2010 U.S. Synthetic Population Quick Start Guide". RTI International. Retrieved from http://www.epimodels.org/midasdocs/SynthPop/2010_synth_pop_ver1_quickstart.pdf, and sources therein.

To generate the synthetic population, RTI used Iterative Proportional Fitting to select households from the 2007–2011 Public Use Microdata Sample (PUMS) data (the 5% sample) to fit marginal distributions of aggregated census counts by census block group. Four matching variables were used to match selected PUMS households to aggregated counts at the block group level—age of the head of household, household income, household size, and race of head of households—as estimated in the 2007–20011 American Community Survey (ACS). To place synthetic households spatially, Integrated Climate and Land Use Scenarios (ICLUS) was used, which provided a population grid at 90-meter resolution.

Workplaces and assignment of workers: Synthetic persons who were over age 18y were assigned to workplaces based on commuting patterns, workplace sizes, and locations. Census data provided commuting patterns of residents, 16 years and older, between census tracts. Using business location shapefiles from ESRI's Business Analyst, the 2010 TIGER Census Tract boundaries, and 2007-2011 Public Use Microdata Area data for employment status of individuals, RTI generated a probability of any one worker commuting from a tract of residence to a tract of business.

Schools and assignment of students: Synthetic persons, who, according to PUMS data, attended school, were assigned to actual schools based on school grade and capacity. RTI examined the person's PUMS school enrollment code (SCH) and school grade level (SCHG) and assigned each person who attended school to the closest school that serviced that grade level. Data sources for assigning students to schools included school locations (HSIP Freedom 2011), and enrollment data (National Center for Educational Statistics (NCES)), in addition to PUMS data.

Table S1: Natural History Parameters

Parameter	Reference Range (mean)	Citation
**Latent Period**	0-2 days (1.4)	Lessler et al, 2009
**Symptomatic Period**	3-6 days (4.7)	Carrat et al, 2008
**Asymptomatic Period**	3-6 days (4.7)	-
**Symptomatic Rate**	0.67	Longini et al, 2004<br>Papenburg et al, 2010
**Asymptomatic infectivity**	0.5	Longini et al, 2004
**Immunity Loss Rate**	0.0	-
**Case-fatality ratio**	0.0	-

Table S2: Contact Parameters

Parameter	Definition	Reference Value	Citation
**Probability of Staying Home**	The baseline probability that an agent stays home if the agent experiences a symptomatic infection.	0.5	Chan, 2007
**Household Contact Probability**	The probability of potentially infective daily contacts between an infectious agent and a susceptible agent in a household.	0.166	calibrated as in (Cooley, 2011)
**Neighborhood Contact Probability**	The probability of potentially infective daily contacts between an infectious agent and a susceptible agent in a neighborhood.	0.000075	calibrated as in (Cooley, 2011)
**School Contact Rates**	The expected number of potentially infective daily contacts between an infectious agent and a susceptible agent in a school	12.4	calibrated as in (Cooley, 2011)
**Workplace Contact Rates**	The expected number of potentially infective daily contacts between an infectious agent and a susceptible agent in a workplace	1.7	calibrated as in (Cooley, 2011)

Table S3. Transmission probability given contact

**Place**	**Infected**	**Susceptible**	**Transmission probability**
Household	Adult	Adult	0.4

Household	Adult	Child	0.3
Household	Child	Adult	0.3
Household	Child	Child	0.6
Elementary School	Student	Student	0.0435
Middle School	Student	Student	0.0375
High School	Student	Student	0.0315
Workplace	Adult	Adult	0.0575
Neighborhood	Adult/Child	Adult/Child	0.0048

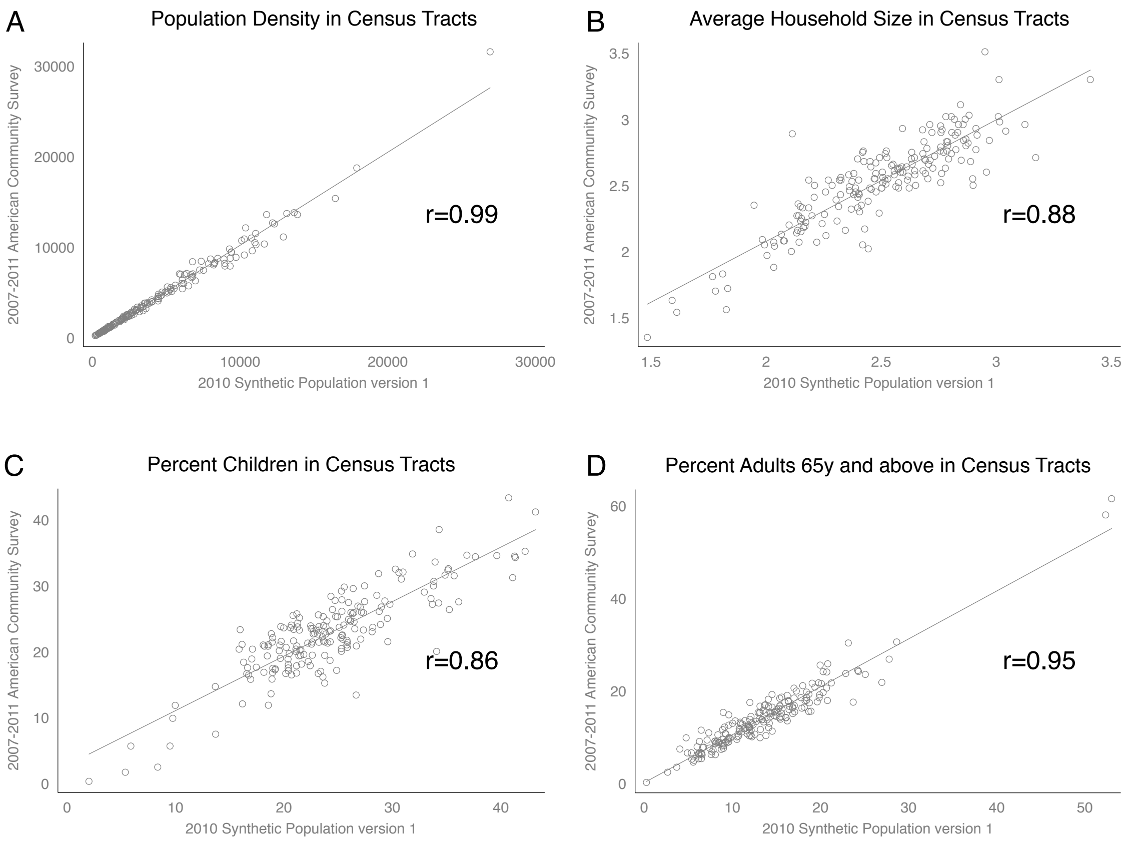

Figure S1. Scatter plots showing correlation between the American Community Survey 2007-11 and the 2010 Synthetic Population version 1 for census tracts within New Haven County, CT. A. Population density in tracts. B. Average Household Size. C. Percent of tract population that is below 18y age. D. Percent of tract population that is 65y or above.

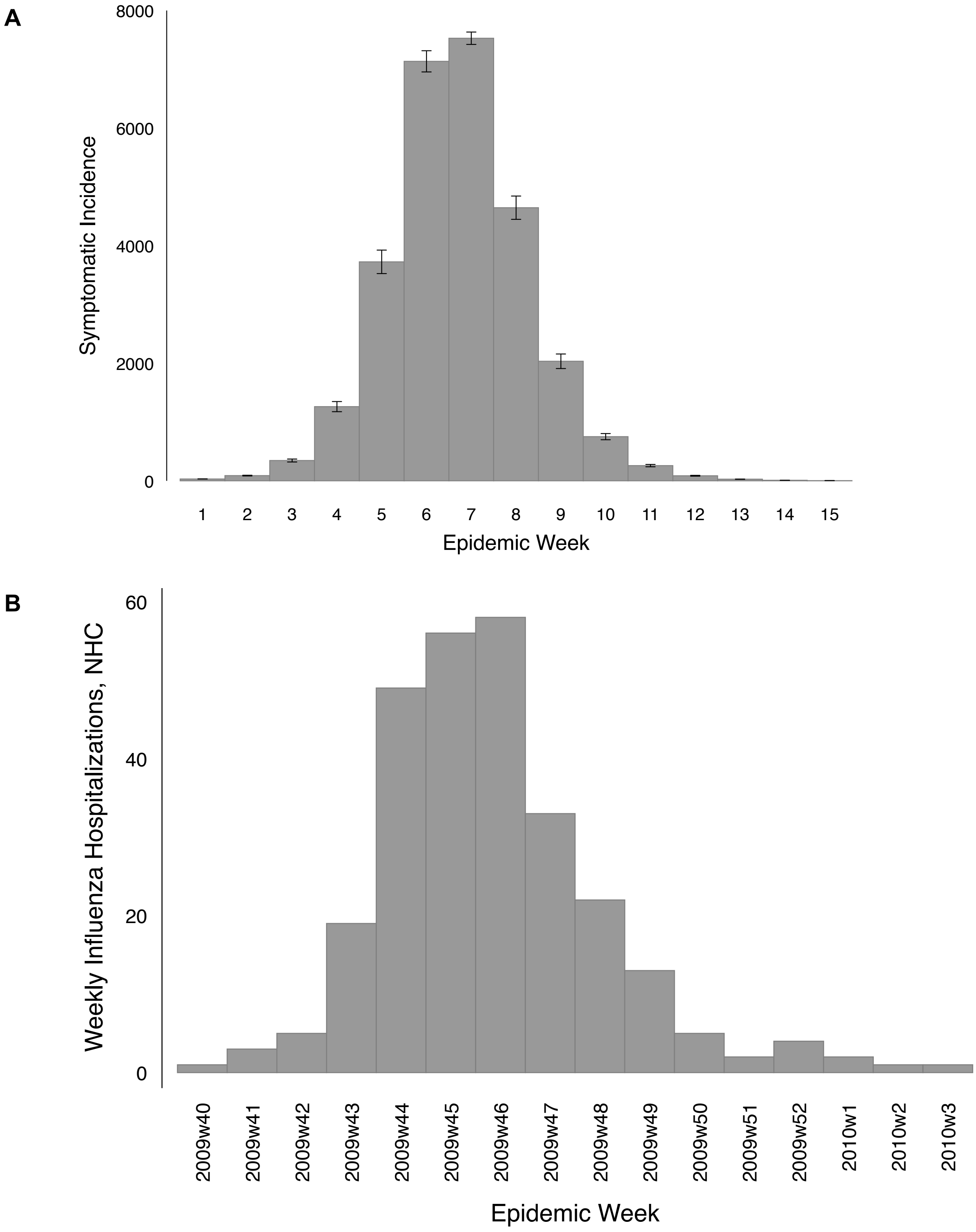

Figure S2. Weekly epidemic curves. From the baseline simulation calibrated to result in a symptomatic AR of 23% (A). From CT-EIP data in NHC showing influenza hospitalized cases (B).

Relationship between poverty and demographic factors

We examined the correlation between demographic factors—population density, average household size, percent of the population below 18y of age and percent of the population above 65y of age—and the percent of the population living below the federal poverty line in the census tract. Poverty was positively correlated with population density ($P < 0.001$) and with the percentage of the census tract population that is below 18y age ($P < 0.001$). Poverty was also negatively correlated with the percentage of older adults in the census tract ($P < 0.001$), but uncorrelated with average household size in the census tract (Figure S3).

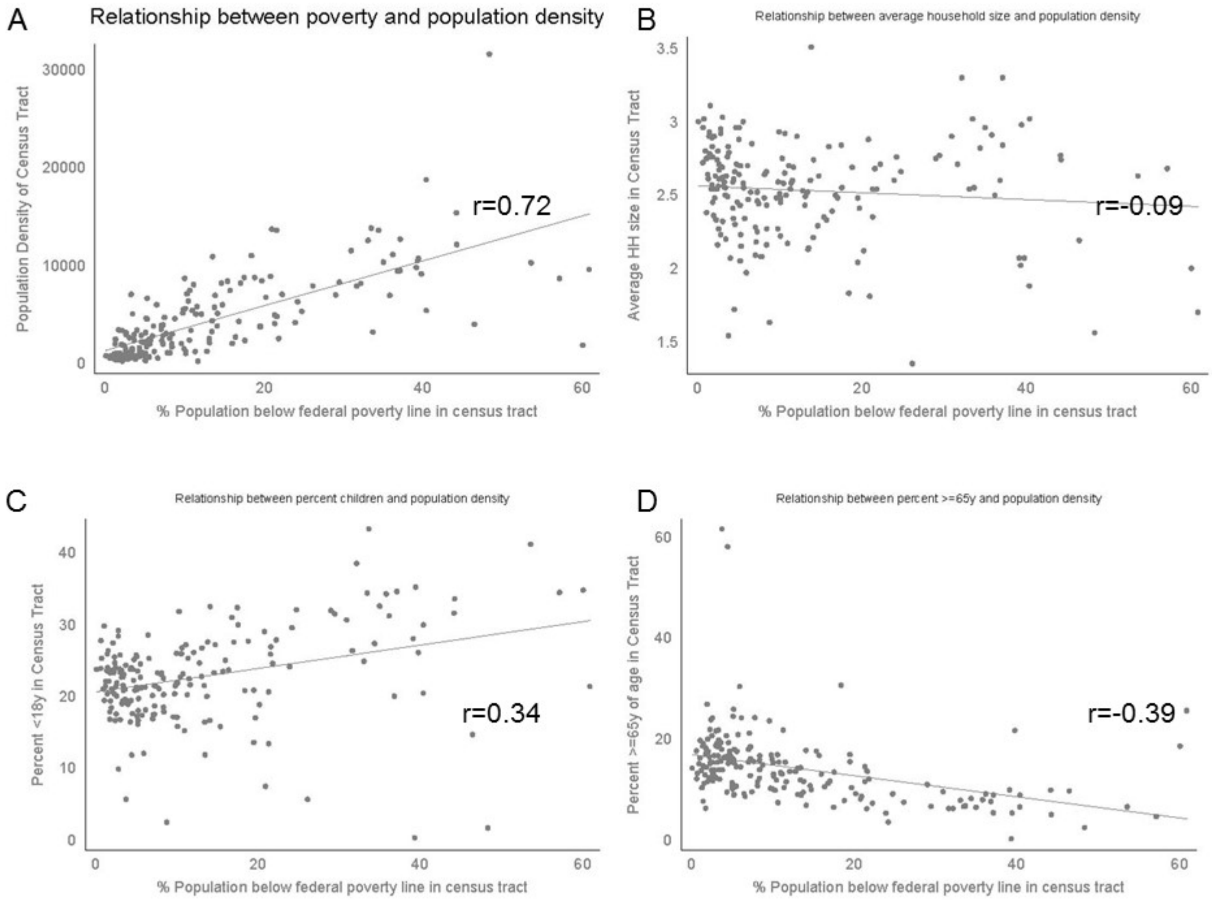

Figure S3. Pearson correlation between the percentage of the population living below the federal poverty line in a census tract and population density in the tract (A), average household size (B), percentage below 18y age (C), and percent >=65y age (D).

Regression analysis

Methods

To examine if factors related to population structure explained the relationship between census tract poverty and AR_{SIM}, we included the percentage of the population in a census tract that was below the federal poverty line in all models along with its squared term to account for the curvilinear relationship between poverty and AR_{SIM}. Population density and its squared term, average household size in a tract, the percent <18y, and the percent >=65y were each included to examine if the relationship between poverty and AR_{SIM} was no longer significant. R^2 was examined to gauge the proportion of variance in AR_{SIM} that was explained by any model. We deemed models to be significant based on the F-test, and model terms to be significant based on the t-test, setting p-value < 0.05 as our cutoff. Beta (standardized) coefficients for each term are reported. We examined the Akaike Information Criterion (AIC; reported in Table S3) as well as the related likelihood ratio test (not shown) to gauge parsimony.

Results

We used multiple regression to examine the demographic factors that account for the relationship between poverty and adult AR_{SIM} in our model. Poverty in a tract explained a little more than a third of the variance in adult AR_{SIM} (Table S3). Once population density and the percent >=65y were included in the regression equation, 46% of the variance in adult AR_{SIM} was explained and the relation between poverty and adult AR_{SIM} was no longer significant. This model was the most parsimonious based on the AIC. Interestingly, the percent >=65y was more important to explaining the relation between poverty and adult AR_{SIM} than the percent <18y. We replaced

percent <18y with percent of the population enrolled in school, and found similar results (not shown).

Table S4. Multiple regression with mean clinical attack rate among adults (AR_{SIM}) as dependent variable and population structure-related factors as explanatory variables. Beta (standardized) coefficients are reported. Proportion of variance explained by each model (R^2), and Akaike Information Criterion (AIC) to compare models are shown.

	**Model Number**						
	M1	M2	M3	M4	M5	M6	M7
% Population living below federal poverty line	**1.39*** **	**0.59***	**1.37*** **	**1.37*** **	**1.06*** **	**0.52***	0.38
% Population living below federal poverty line (squared term)	**-0.93*** **	**-0.46***	**-0.92*** **	**-0.92*** **	**-0.71*** **	**-0.43***	-0.31
Population density in census tract (per sq. mi.)		**0.74*** **				**0.77*** **	**0.69*** **
Population density in census tract (squared term)		**-0.31***				**-0.30***	**-0.31***
Average household size			**0.14***			**0.18****	
% <18y				0.02			
% >=65y					**-0.29*** **		**-0.25*** **
Adjusted R^2	34%	42%	36%	34%	40%	45%	46%
AIC	1116	1095	1111	1117	1098	1086	*1080*

********P* **< 0.001; *****P* **< 0.01; ****P* **< 0.05.**

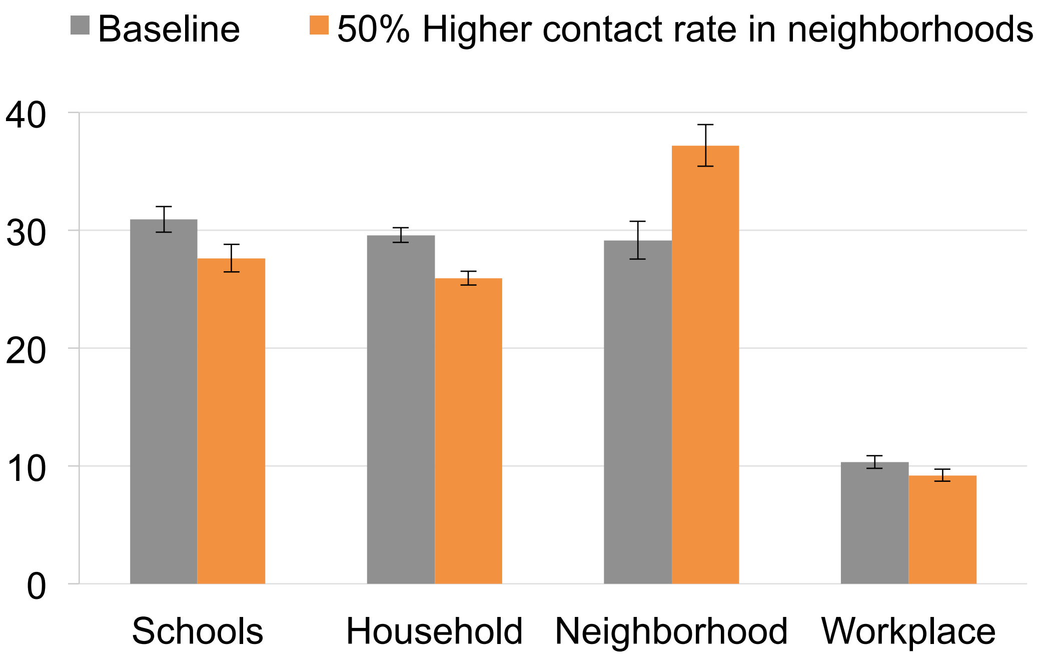

Figure S4. Place-based infection percentages in a scenario with calibrated neighborhood contact rates (baseline; gray) or 50% higher contact rates in the neighborhood compared to baseline (orange).

Household income-based susceptibility

Additional Methods

We examined the weekly simulated epidemic curve for each household income-based susceptibility scenario, and note peak week, peak height, and outcomes in Table S5.

We examined possible interaction between higher neighborhood contact rates and differential susceptibility to disease by running models in which susceptibility of highest-income households was 77%, 50%, and 14% that of lowest-income households and agents had 20% higher contacts in the neighborhood compared to the calibrated value.

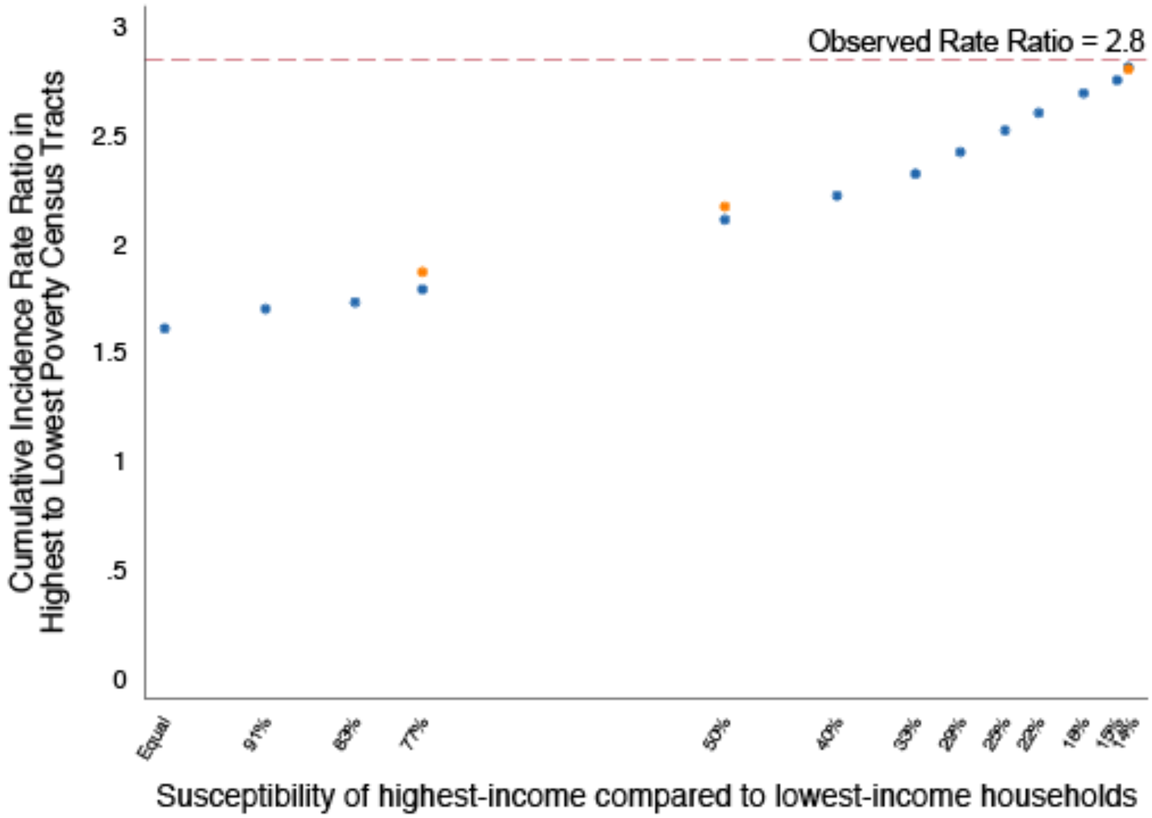

Figure S5. Income-based difference in susceptibility impacts area-level disparities. The red dashed line represents the observed hospitalization rate ratio between the highest and lowest poverty tracts in NHC. Blue dots represent model-generated ratios as susceptibility of high-income households declined in comparison with low-income households. Orange dots represent model-generated ratios with 20% higher contacts in the neighborhood compared to the calibrated contact rate.

Table S5. Comparison of epidemic characteristics under different household income-based susceptibility conditions

Susceptibility ratio (High/Low income)	Peak Week	Peak height (symptomatic incidence)	Length of outbreak (weeks)	Overall AR_{SIM}	RR_{SIM} (Ratio of AR_{SIM} in highest to lowest poverty level)
1 (Baseline)	7	7528	15	23	1.61
0.91	7	7350	15	22	1.70
0.83	7	7507	15	23	1.73
0.77	7	7474	15	23	1.79
0.50	6	7343	15	22	2.11
0.40	6	8668	15	23	2.22
0.33	6	8600	15	23	2.32
0.29	6	8195	15	23	2.42
0.25	6	7965	15	22	2.52
0.22	6	7724	15	22	2.60
0.18	5	8656	15	22	2.69
0.15	5	9109	15	22	2.75
0.14	5	8878	15	21	2.81
